# Salvianolic Acids Attenuate Rat Hippocampal Injury after Acute CO Poisoning by Improving Blood Flow Properties

**DOI:** 10.1155/2015/526483

**Published:** 2015-02-01

**Authors:** Li Guan, Yan-Lin Zhang, Zong-Yang Li, Ming-Xia Zhu, Wei-Juan Yao, Jin-Yuan Zhao

**Affiliations:** ^1^Research Center of Occupational Medicine, Peking University Third Hospital, 49 North Garden Road, Haidian District, Beijing 100191, China; ^2^Hemorheology Center, School of Basic Medical Sciences, Peking University Health Science Center, 38 Xue Yuan Road, Haidian District, Beijing 100191, China

## Abstract

Carbon monoxide (CO) poisoning causes the major injury and death due to poisoning worldwide. The most severe damage via CO poisoning is brain injury and mortality. Delayed encephalopathy after acute CO poisoning (DEACMP) occurs in forty percent of the survivors of acute CO exposure. But the pathological cause for DEACMP is not well understood. And the corresponding therapy is not well developed. In order to investigate the effects of salvianolic acid (SA) on brain injury caused by CO exposure from the view point of hemorheology, we employed a rat model and studied the dynamic of blood changes in the hemorheological and coagulative properties over acute CO exposure. Compared with the groups of CO and 20% mannitol + CO treatments, the severe hippocampal injury caused by acute CO exposure was prevented by SA treatment. These protective effects were associated with the retaining level of hematocrit (Hct), plasma viscosity, fibrinogen, whole blood viscosities and malondialdehyde (MDA) levels in red blood cells (RBCs). These results indicated that SA treatment could significantly improve the deformation of erythrocytes and prevent the damage caused by CO poisoning. Meanwhile, hemorheological indexes are good indicators for monitoring the pathological dynamic after acute CO poisoning.

## 1. Introduction

Carbon monoxide (CO) intoxication is the most common type of poisoning worldwide [1]. CO is a colorless, nonirritating, odorless, and tasteless gas. Despite received various forms of treatments, forty percent of survivors develop delayed encephalopathy after a lucid interval from a few weeks up to months from the acute poisoning. The symptom includes inappropriate euphoria, impaired judgment, poor concentration, memory loss, cognitive and personality changes, psychosis, and Parkinsonism symptoms. A cohort analysis of 256 patients with carbon monoxide poisoning suggests that the severity of the initial symptoms is not correlated to the delayed-onset effects [2]. In addition, neuronal apoptosis, necrosis, and oxidative stress occur within short period (hours or days) after CO poisoning [3–6]. Prevention of these pathologic processes can reduce brain damage and improve the functional recovery and survival rate [7, 8]. Unfortunately, the etiology of delayed encephalopathy after acute CO poisoning (DEACMP) remains unclear.

DEACMP happens more often in the elders with cardiovascular or cerebrovascular diseases [9]. Furthermore, the regions with poor vasculatures in brain such as white matter and basal ganglia are also very sensitive to hypoxic damage [10, 11]. This association of vasculature properties and DEACMP indicates that the disturbance of cerebral circulation may have a high impact on the induction of delayed cerebral injury after CO exposure.

Hemorheological parameters are the most important vasculature properties. Hemorheology is an important subject that studies the flow properties of blood circulation and its elements (plasma and formed elements, including red blood cells, white blood cells, and platelets). Whole blood viscosity and erythrocyte aggregation and deformation are key hemorheological parameters [12]. Collective evidences indicate that the flow properties of blood are among the main determinants of proper tissue perfusion and the alterations, which play significant roles in many disease processes [13–17]. It has been shown that hemorheological derangement (i.e., whole blood, plasma, and serum hyperviscosity, reduced erythrocyte deformability, increased red cell aggregation, and hyperfibrinogenemia) can potentially impair micro- and macrovascular blood flow in the brain of Alzheimer's disease subjects [18]. The hemorheologic changes are also important factors in determining the clinical outcomes for the patients with coronary or cerebral arterial diseases [19–21]. We hypothesize that the development of DEACMP may be associated with the deterioration of hemorheological parameters after CO exposure. Therefore, in the present study, we investigated the relationship between DEACMP and hemorheologic dynamic changes.

Salvianolic acid (SA) is commonly used in clinical trials to decrease blood viscosity. SA is a natural product isolated from the Chinese traditional medicine,* Salvia miltiorrhiza*. Besides improving the blood circulation [22], SA is also a strong antioxidant with the activity of inhibiting lipid peroxidation, scavenging hydroxyl radical, preventing platelet aggregation, and so forth [23, 24].

In this study, we employed a rat model to investigate the dynamic of hemorheological changes at different time after acute CO poisoning. We found that the treatment of SA significantly improved the hemorheological properties and, more importantly, prevented the neuronal damage. This indicates that the improvement of blood flow properties could indeed relieve the neuronal damage in CO poisoning. This study provides the new insight of CO toxicology to central nervous system effects in perspectives of hemorheology and the administration of SA to prevent of DEACMP.

## 2. Materials and Methods

### 2.1. Animals

Male Sprague-Dawley rats (300–350 g) were obtained from the Peking University Animal Breeding Unit. All animals were raised under the same laboratory conditions of temperature (25°C) and lighting (12/12 h light/dark cycle) and were given free access to standard laboratory chow and tap water. All rats were allowed to acclimatize for 1 week prior to experimentation. All experimental procedures involving animals were approved by the Ethical Animal Committee of Peking University, Beijing, China.

### 2.2. Reagents

Carbon monoxide was purchased from Beijing Beiyang Special Institute Co. (China); SA was supplied by the Modern Research Center for Traditional Chinese Medicine of Peking University. PVP- (polyvinyl pyrrolidone-) K30 was provided by Shanghai Chemical Instrument Co. MDA assay kit was purchased from Nanjing Jiancheng Agent Co. All other chemicals were of analytical grade from commercial suppliers.

### 2.3. Treatments

All substances were dissolved in pyrogen-free saline unless indicated otherwise. Experimental animal groups were as follows. (1) CO exposure group (*n* = 15): CO poisoning (more than 70% carboxyhemoglobin in the blood) was induced in rats by exposure to air supplemented with 3000 ppm CO introduced into the chamber at a flow rate of 8 L/min for 40 min [25, 26]. (2) Control group (*n* = 25): rats were treated with air by the same method. (3) Salvianolic acids (SA) + CO exposure (*n* = 25): SA (6 mg/kg body weight /day) was administered to rats every day via the tail vein after CO poisoning [27]. (4) 20% mannitol + CO exposure (*n* = 25): 20% mannitol (10 mL/kg body weight) was administered every day via the tail vein after CO treatment [28]. The blood samples were collected from eye socket at 1 h, 1–5 days after CO exposure for detection of various hemorheological parameters. Rats were sacrificed at day 5 and their brains were removed for histochemical stains. The rats had free access to drinking water during the experimental period.

### 2.4. Histochemical Staining and Counting of Necrotic Neurons

For analysis of hippocampal neuronal survival, rats that survived for 5 days were deeply anesthetized with halothane, perfused through the heart with approximately 200 mL phosphate-buffered saline (0.05 M sodium phosphate pH 7.2, 0.1 M NaCl), and perfusion was fixed with 4% paraformaldehyde. Brains were removed and fixed in 4% paraformaldehyde before paraffin embedding and dissection. Serial sections (5 *μ*m) were cut (corresponding to bregma −3.3 cm according to the atlas of Paxinos and Watson 11) and stained with hematoxylin and eosin (H&E). Viable pyramidal neurons were counted in the CA1 region of the hippocampus under a high-power field (×400). Necrotic neurons were recognized by their pyknotic or karyorrhectic nuclei lacking clear nucleoli, whereas viable neurons were defined as those cells showing distinct nuclear and nucleolar structures. The number of normal pyramidal neurons per high-power field was counted by an observer blinded to the experimental protocol.

### 2.5. Hematological Analysis and Measurement of Plasma, Whole Blood Viscosities, and Fibrinogen Concentration

Heparinized blood samples were drawn from eye socket of rats 1 h, 1~5 days after CO exposure. Twenty microliters of fresh blood was applied to a full-automated hematological analyzer (Sysmex KX-21N, Japan) for determination of hematocrit (Hct). Plasma was separated by centrifuging blood at 150 g. The plasma viscosity and whole blood viscosity at different shear rates (200, 50, and 5 s^−1^) were measured with an automatic viscometer (LG-R-80B, Steellex Co., Beijing, China). Fibrinogen concentration was measured using coagulant analyzer (RABRE, Steellex Co., Beijing, China).

### 2.6. Measurement of Deformation Index and Aggregation Index of Red Blood Cells (RBCs)

Red blood cells (RBCs) were suspended in 15% PVP buffer (w/V, MW = 30 kDa, 61 mM NaCl, 0.8 mM Na_2_HPO_4_, pH 7.4, 290 mOsm/kg, and viscosity 15 mPa s) and adjusted to the concentration of 2 × 10^7^/mL. Bovine serum albumin (BSA) of 40 mg/mL was added into the buffer to keep the morphology of RBCs. The deformation indices (DI) at shear rates of 50–1000 s^−1^ were measured with a traditional ektacytometer (Model LBY-BX2, Precil Co., Beijing, China) [29]. Five hundred microliters of blood was used for RBC aggregation measurement with a photometric aggregometer (Steellex Co., Beijing, China) interfaced to a computer. The cells were sheared at 1000 s^−1^ for 5 s. After the shearing, the change of light transmission through RBC suspension was monitored and an aggregation rate was calculated.

### 2.7. Estimation of Lipid Peroxidation of RBCs

MDA determination is based on spectrophotometric or spectrofluorimetric measurement of the condensation product formed from MDA and 2-thiobarbituric acid (TBA) by measuring the absorbance at 532 nM after reaction.

### 2.8. Statistical Analyses

The data is expressed as mean ± SD. Statistical analyses were performed in SPSS13.0 software. The differences between groups were analyzed by ANOVA and considered to be statistically significant when *P* < 0.05.

## 3. Results

### 3.1. The Pathological Changes in Hippocampus

**Figure 1:**
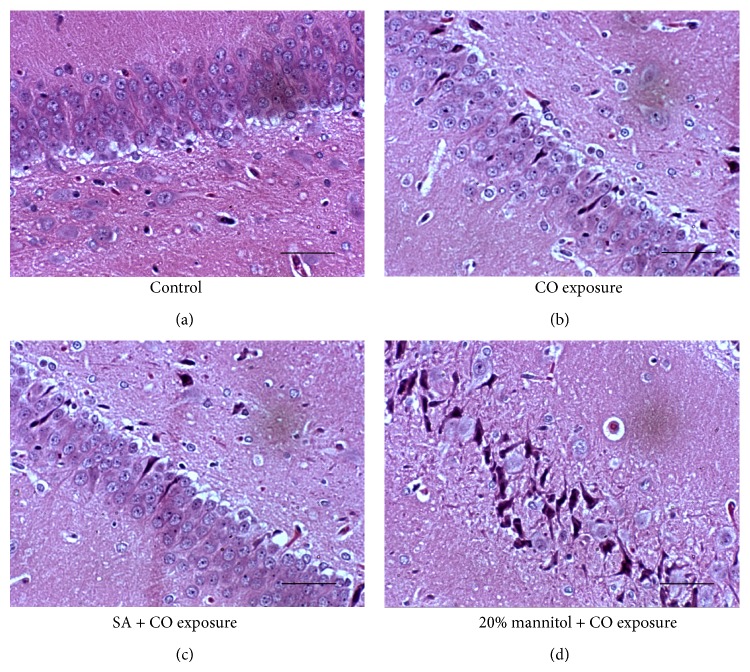
Neuronal survival and necrosis in the CA1 region of CO-exposed rates. The panels show representative H&E stained sections from control group (a), the CO exposure group (b), the SA + CO group (c), and the 20% mannitol + CO group (d). (a) The control rats, with rare neuronal death occurring in the CA1 region; (b) CO-exposed rats, with massive neuronal degeneration, apoptosis, or necrosis, and neuronal density decreased, as arrows indicated; (c) SA + CO group rats: neuronal density and cell morphology were well preserved; only mild degeneration of neuron could be observed occasionally; (d) 20% mannitol + CO exposure rats: severe damage of neuron was observed in CA1 region which was more serious than CO-exposed alone rats, as arrows indicated. All images were captured at 400x magnification; calibration bar, 100 *μ*m.

Brain tissue sections from each group were H&E stained and examined under the light microscope on day 5. In CO group and 20% mannitol + CO group, the hippocampus displayed an abnormal histology, with marked changes in the thickness of the pyramidal cell layers (Figure 1). The numbers of neurons in the CA1 subfield were evaluated by counting the viable neurons in each field. The neuron densities reduced by 29% and 63%, respectively, in animals treated with CO or CO + 20% mannitol (Figure 1 and Table 1). In SA + CO group, however, the number of surviving neurons (72 ± 9 per field) was significantly higher than CO group (58 ± 10 per field). The difference between SA + CO group and the control group was not statistically significant (*P* > 0.05).

### 3.2. Characteristics of Hematological Parameters in CO-Poisoned Rats

**Figure 2:**
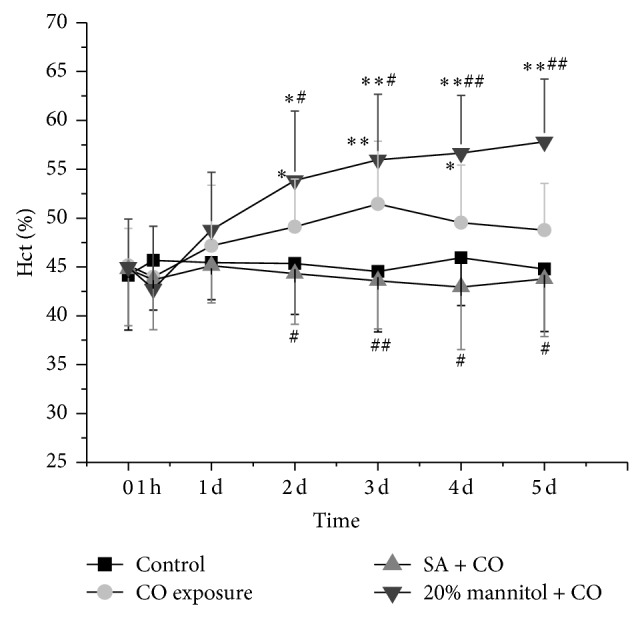
Comparison of Hct among the CO exposure group, the SA + CO group, the 20% mannitol + CO group, and the control group. ^*^ *P* < 0.05 and ^**^ *P* < 0.01 versus control group. ^#^ *P* < 0.05 and ^##^ *P* < 0.01 versus CO exposure group.

The hematocrit (Hct) changes after CO exposure were presented in Figure 2. In CO group, Hct slightly dropped at 1 h and started to increase from day 1 (*P* < 0.05 compared to control) and reached the highest level on day 3 (*P* < 0.01). There was a little drop afterwards but it was still higher than control (*P* < 0.05). In 20% mannitol + CO group, Hct was significantly elevated compared to CO group from day 1 (*P* < 0.01). In SA + CO group, the elevation of Hct was greatly prevented by SA infusion.

**Figure 3:**
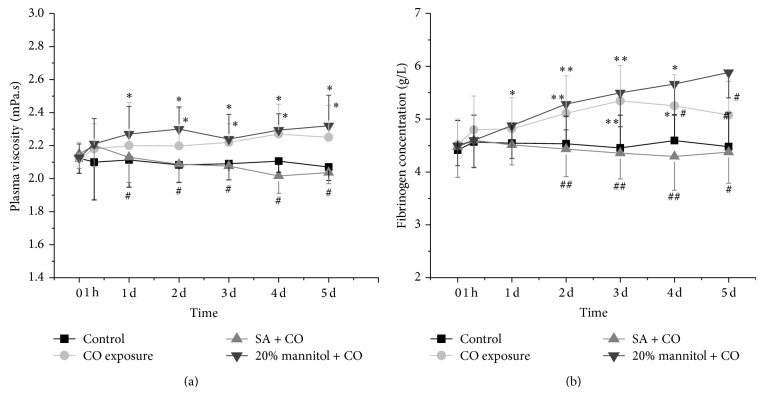
Comparison of plasma viscosity and fibrinogen concentration among the CO exposure group, the SA + CO group, the 20% mannitol + CO group, and the control group.^*^ *P* < 0.05 and ^**^ *P* < 0.01 versus control group. ^#^ *P* < 0.05 and ^##^ *P* < 0.01 versus CO exposure group.

The plasma viscosities and fibrinogen concentrations in different groups were detected (Figures 3(a) and 3(b)). The plasma viscosities and fibrinogen concentrations rose at 1 hour and kept increasing to the highest level on day 3 (*P* < 0.05 (Figure 3(a)); *P* < 0.01 (Figure 3(b))) in both CO and 20% mannitol + CO groups. On day 5, both of them still remained higher than control. No significant difference in Hct, plasma viscosity, and fibrinogen concentration between SA + CO group and control was observed on days 1–5.

### 3.3. The Changes in Whole Blood Viscosity

**Figure 4:**
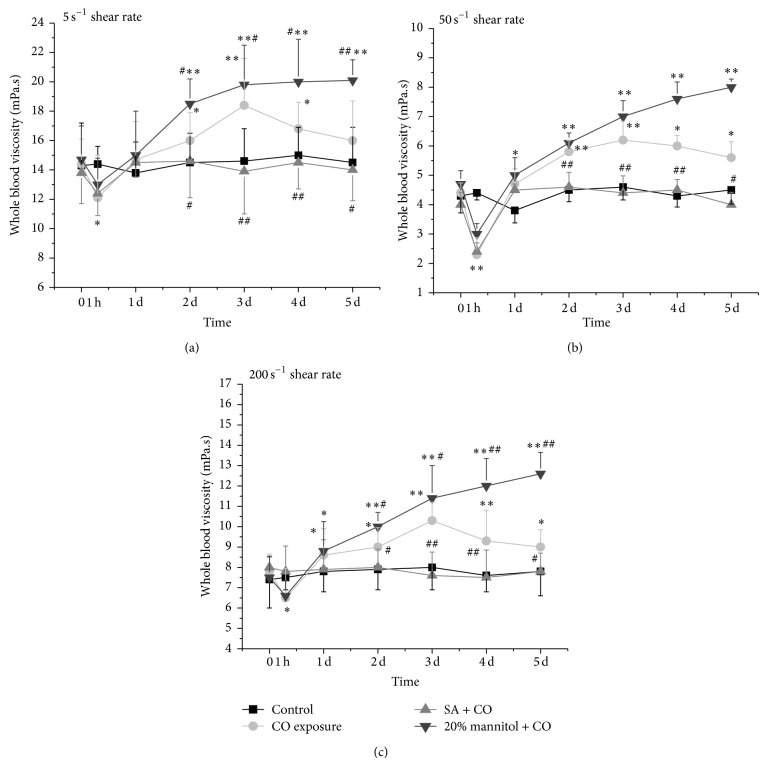
Comparison of whole blood viscosities at high, medium, and low shear rates among CO exposure group, SA + CO group, 20% mannitol + CO group, and control group. ^*^ *P* < 0.05 and ^**^ *P* < 0.01 versus control group. ^#^ *P* < 0.05 and ^##^ *P* < 0.01 versus CO exposure group.

The whole blood viscosities at high (200 s^−1^), medium (50 s^−1^), and low (5 s^−1^) shear rate showed similar pattern (Figures 4(a), 4(b), and 4(c)). They all decreased significantly (*P* < 0.01) at 1 hour after CO exposure. But, 1 day later, they began to increase and recovered to the control level on day 5 in CO group. While in 20% mannitol + CO group the whole blood viscosities kept high level until day 5 (*P* < 0.01), with SA + CO treatment, the whole blood viscosities recovered to control level 1 day after CO exposure and remained at a similar level to that of control group.

### 3.4. Changes of the Deformability and Aggregation of Erythrocytes

**Figure 5:**
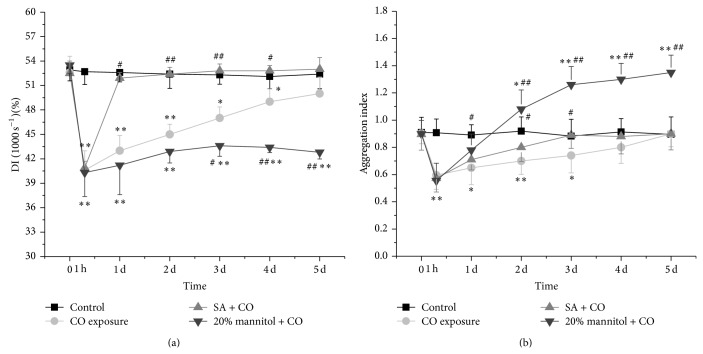
Comparison of deformability and aggregation of erythrocytes among the CO exposure group, the SA + CO group, the 20% mannitol + CO group, and the control group. ^*^ *P* < 0.05 and ^**^ *P* < 0.01 versus control group. ^#^ *P* < 0.05 and ^##^ *P* < 0.01 versus CO exposure group.

The deformation index (DI) and aggregation index were measured and the data were shown in Figures 5(a) and 5(b). DI decreased at 1000 s^−1^ at 1 hour after CO exposure (*P* < 0.01). It slightly increased on day 1 and gradually reached the control level on day 5 in CO group. In SA + CO group, DI obviously increased on day 1 when compared with CO and 20% mannitol + CO groups (*P* < 0.01). There was no significant difference between control group and SA + CO group from day 1 to day 5.

The aggregation index of CO group dropped significantly at 1 hour after CO exposure and started to recover on day 1. Finally it approached the normal level by day 5. In SA + CO group, the increase in aggregation index was more significant than the CO group from day 1 (*P* < 0.05). But, in 20% mannitol + CO group, the aggregation index began to greatly increase from 0.55 ± 0.13 to 1.35 ± 0.12 on day 1 and remained high till day 5.

### 3.5. Changes of MDA in Erythrocytes

**Figure 6:**
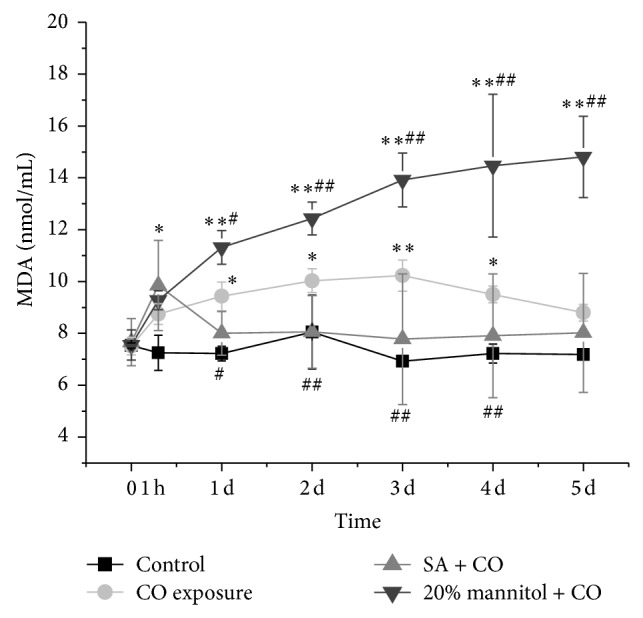
Comparison of MDA level among the CO exposure group, the SA + CO group, the 20% mannitol + CO group, and the control group. ^*^ *P* < 0.05 and ^**^ *P* < 0.01 versus control group. ^#^ *P* < 0.05 and ^##^ *P* < 0.01 versus CO exposure group.

The MDA levels in erythrocytes were measured and shown in Figure 6. The MDA levels kept increasing along with the treatment in CO group (*P* < 0.01), while in SA + CO group MDA levels were lower than those in CO and 20% mannitol + CO groups from day 1 (*P* < 0.05), despite that they were still slightly higher than those in the control group.

## 4. Discussion

In the present study, we found that CO poisoning caused the deterioration of hemorheological properties and severe neuronal damage in rats. The treatment of SA significantly improved the blood flow and coagulant properties and, more importantly, prevented the neuronal damage.

Normal deformability of RBCs, also known as erythrocyte deformability, is an important property for the transportation of oxygen in blood as well as the normal perfusion of microcirculation [30, 31]. Decreased deformability of RBCs may lead to a reduction of life span of RBCs as well [32]. Cicco and Pirrelli reported that as low as 10% decrease in erythrocyte deformability in hypertensive patients might lead to a significant impairment in both cellular and tissue oxygen delivery [33]. Our study showed that DI decreased after CO exposure, reflecting a definite change in deformability of RBCs. Erythrocyte deformability in CO and 20% mannitol + CO groups decreased about 28% and 53%, respectively. These indicated that there were dysfunctions in cellular oxygen delivery and tissue oxygen delivery in CO-exposed animals. Furthermore, this change can be prevented by the application of SA. Erythrocyte deformability was improved nearly 20% in SA + CO group, which suggested the therapeutical potential of SA administration for CO poisoning.

Our study also showed MDA level in RBCs, which is one of the most important ending products of lipid peroxidation. MDA presented an abrupt increase at the very beginning of CO exposure and remained high till the end of the experiment (5 days after CO exposure). This data suggests there was an enhancement of lipid peroxidation after CO exposure. The corresponding oxidant stress may decrease the erythrocyte deformability in the early stage. Lipid peroxidation may or may not be the direct reason for the impairment of erythrocyte deformability, because the membrane protein components, the major determinants for RBC mechanical properties, are independent upon lipid peroxidation. Condon et al. found that depletion of white blood cell (WBC) was sufficient to abrogate the ability of trauma hemorrhagic shock (T/HS) lymph to cause abnormal RBC deformability, indicating that RBC injury by T/HS lymph requires WBC [34]. It is likely that the enhanced oxidant stress induced polymorphonuclear neutrophils (PMN) respiratory burst activity. As a result, multiple mediator networks were stimulated, such as complements, kinins, coagulation, and fibrinolysis cascades, along with the release of chemokines, cytokines, soluble receptors, lipid mediators, and numerous enzymes such as elastase, myeloperoxidase, and proteases. The net result could lead to the changes of RBC hemorheology properties and various pathological abnormalities after CO exposure.

Another important property of RBCs is the ability to aggregate. In large vessels, RBC aggregates flow in the center of the vessels, which create a cell-free layer near the vessel wall, thereby facilitating flow. In smaller vessels, the flow-induced shear stress disperses the aggregates so RBCs can flow through the capillaries [35, 36]. It is known that erythrocytes tend to aggregate with lower velocities of circulating blood, and erythrocyte aggregation may contribute to the margination and adhesion of leukocytes and platelets to the vessel wall [37]. Erythrocyte aggregation is one of the key factors of whole blood viscosity at low shear rates [38]. When RBC aggregability is enhanced, the aggregates could obstruct capillary blood flow and inhibit tissue oxygenation. Yet, in this study, we found that erythrocyte aggregation decreased rapidly right after CO exposure (Figure 5(b)) and it returned to the control level on day 4. The decreased erythrocyte aggregation may contribute to the reduction of whole blood viscosity at low shear rates 1 h after intoxication but may not be beneficial for leukocyte and platelets adhesion. The reason that caused RBC aggregation change should be further studied.

SA blocked the CO-induced increase in hippocampal injury and improved the hemorheological parameters, including Hct, plasma viscosity, fibrinogen, and whole blood viscosities. In response to CO exposure, the whole blood viscosities dipped in a quick fashion and recovered 24 hours later (Figure 4), while in CO and 20% mannitol + CO groups the whole blood viscosities not only returned to the normal value but also continued increasing till 5 days after the exposure (Figure 5). CO causes lipid peroxidation by releasing nitric oxide (NO) and oxygen-free radicals from endothelial cell and platelet [39]. Therefore, a compensative and protective reaction to hypoxia in microcirculation perfusion after CO exposure might happen. There are several interpretations based on this observation. The widely accepted view is that the increase in blood flow may be able to supply enough oxygen so that there is no change in the oxygen consumption. Another interpretation is that the increase in blood flow may cause vasodilation independently based upon oxygen level with dilatory agent (such as NO) and maintain adequate tissue oxygen level with the increased oxygen extraction [40]. But the change in whole blood viscosity at low shear rate occurring on day 1 after CO exposure could have disturbed the microcirculation in brain, especially in the regions with poor vasculatures, such as white matter, globus pallidus, basal ganglia, and hippocampus. The constant increase in plasma fibrinogen and changes with hematocrit after 2 days may contribute to the increase in whole blood viscosity (Figures 2 and 3).

## 5. Conclusion

Our study first reported that there were hemorheological changes in brain after acute CO exposure. The decrease in the whole blood viscosity as a compensatory reaction at early stage may contribute to accelerating brain blood flow and improving oxygen cerebral supply. But the increase in whole blood viscosity may affect the blood perfusion in heart and cerebral microcirculations and even result in ischemia especially in the regions with poor vasculature such as white matter, globus pallidus, basal ganglia, and hippocampus. The study also showed that SA could efficiently clean out the free radicals, well improve the deformability of RBCs after CO exposure, and greatly relieve hypoxemia and pathological abnormalities of hippocampus.

## Figures and Tables

**Table 1:** The numbers of damaged neurons in the hippocampal CA1 region of rats in different groups.

Hippocampal region	Group			
	Control	CO exposure	SA + CO exposure	20% mannitol + CO exposure
CA1 subfield	82 ± 7	58 ± 10^*^	72 ± 9^#^	30 ± 4^∗/#^

*Note*. Data are means ± SEM; ^*^ *P* < 0.01 versus control; ^#^ *P* < 0.01 versus CO exposure.
